# Real-world evidence on methotrexate-free subcutaneous tocilizumab therapy in patients with rheumatoid arthritis: 24-week data from the SIMPACT study

**DOI:** 10.1093/rap/rkac038

**Published:** 2022-05-16

**Authors:** György Nagy, Pál Géher, László Tamási, Edit Drescher, Péter Keszthelyi, Judit Pulai, László Czirják, Zoltán Szekanecz, Gergely Kiss, László Kovács

**Affiliations:** 1 Department of Rheumatology & Clinical Immunology, Department of Internal Medicine & Oncology; 2 Department of Genetics, Cell & Immunobiology; 3 Heart and Vascular Center, Semmelweis University, Budapest; 4 Department of Rheumatology, BAZ County Central Hospital, Miskolc; 5 Department of Rheumatology, Csolnoky Ferenc Hospital, Veszprém; 6 Department of Rheumatology, Békés County Central Hospital, Gyula; 7 Department of Rheumatology, Fejér County Saint George Hospital, Székesfehérvár; 8 Department of Rheumatology & Immunology, University of Pécs Clinical Centre, Pécs; 9 Department of Internal Medicine, Rheumatology, University of Debrecen Clinical Centre, Debrecen; 10 Roche (Hungary) Ltd, Budapest; 11 Rheumatology & Immunology Clinic, University of Szeged Clinical Centre, Szeged, Hungary

**Keywords:** rheumatoid arthritis, tocilizumab, monotherapy, subcutaneous, steroid, DAS28, clinical activity index

## Abstract

**Objectives:**

The aim of the SIMPACT study was to evaluate the efficacy and safety of MTX-free s.c. tocilizumab (TCZ) therapy in RA patients.

**Methods:**

SIMPACT was an open-label, non-controlled, non-randomized, non-interventional study, in which RA patients for whom the treating physicians ordered s.c. TCZ were observed during a 24-week treatment period in Hungarian centres. Although the use of MTX was avoided during the study period, other conventional synthetic DMARDs, oral CSs and NSAIDs were allowed. Study endpoints included the change in DAS28 and clinical activity index (CDAI) scores, the proportion of patients achieving remission in the whole population and in subgroups defined based on prior RA treatment history, and age, weight or biological sex *post hoc*. The extent of supplementary medication use was monitored.

**Results:**

Three hundred and thirty-seven RA patients were enrolled in 18 study centres. TCZ therapy significantly decreased the disease activity measured by both DAS28 (*P *=* *0.0001) and CDAI (*P *=* *0.0001). Clinical response was more pronounced in biologic-naïve patients and was lower in patients >75 years of age. In the whole population, DAS28 ESR or CRP and CDAI remission rates were 70.10%, 78.95% and 33.59%, respectively. In patients <45 years of age, the CDAI remission rate doubled (67.86%). A significant decrease in the frequency of co-administered medication was reported, including oral CSs and DMARDs.

**Conclusion:**

Real-world clinical evidence on s.c. TCZ reported here is in line with the efficacy outcomes of randomized clinical trials. Subgroup analysis revealed that TCZ was more effective in biologic-naïve patients and in those <75 years old.

**Trial registration:**

ClinicalTrials.gov, http://www.clinicaltrials.gov, NCT02402686.

Key messages

- Real-world evidence of s.c. tocilizumab therapy parallels randomized clinical trials, even without MTX co-administration.
- Tocilizumab s.c. therapy was more effective in biologic-naïve patients and showed an age-related decrease in efficacy.
- The frequency of co-administered oral CSs and DMARDs was significantly decreased during the study period.

## Introduction

IL-6 is a pleiotropic pro-inflammatory cytokine involved in diverse physiological processes that plays a central role in the pathogenesis of RA. Tocilizumab (TCZ), a humanized mAb against the IL-6 receptor, has proven efficacy in treating RA upon both i.v. and s.c. administration [1–5].

Practice-based studies demonstrate that biologics, including TNF inhibitors, are more effective in combination with MTX [6, 7]. To improve the efficacy of biologic DMARDs, both EULAR recommendations and ACR guidelines recommend supplementation of the biologic DMARDs with conventional synthetic DMARDs, such as MTX [8, 9]. However, when combination with MTX or other conventional synthetic DMARDs is not possible owing to contraindications or intolerance, some biologics might be used as monotherapy [10, 11]. It is noteworthy that, based on real-world data, approximately one-third of RA patients who require biologic treatment in clinical practice receive a biologic DMARD as monotherapy (without MTX) [12–14]. The efficacy of s.c. TCZ as monotherapy in RA patients with active disease was previously established in randomized clinical trials (RCTs) [15–17]; however, real-world data are sparse.

The objective of the present non-interventional, real-world clinical study (SIMPACT) was to evaluate the efficacy of s.c. TCZ in patients with RA in a standard of care setting for whom MTX is contraindicated. Considering that only a subpopulation of RA patients treated with biologic DMARDs is eligible for major clinical trials, comparison of the results observed in daily clinical practice with the results reported from RCTs is of particular interest [18–20].

## Methods

### Study design and patients

This open-label, non-randomized, single arm, multicentre, observational study was designed to collect real-world efficacy and safety data. Adult male or female patients with a diagnosis of moderate to severe RA based on the ACR/EULAR classification criteria [21] and with an established MTX contraindication/intolerance, for whom s.c. TCZ therapy was commenced within 8 weeks before the enrolment according to standard of care and in line with the current Summary of Product Characteristics or local labelling, were eligible for participation. All participants gave written informed consent. Patients with a history of systemic (except SS) or organ-specific autoimmune diseases (including Hashimoto thyroiditis) or joint inflammatory disease other than RA were excluded from the study. Additional exclusion criteria included prior s.c. or i.v. TCZ treatment, and treatment with any investigational drugs 30 days before enrolment. Patients did not use MTX during the entire study period, and any treatment with MTX <1 week before TCZ initiation led to exclusion from the study.

### Treatment

Patients were treated according to everyday clinical practice in line with the relevant therapeutic recommendations and protocols. No new diagnostic or therapeutic options were tested in this non-interventional study. According to the current label, eligible subjects were projected to receive 162 mg/week s.c. TCZ injection for 24 weeks. Although MTX was omitted, treatment with other medication, including conventional synthetic DMARDs or oral CSs and NSAIDs, was allowed and observed during the study. The choice of therapy was based exclusively on the medical decision of the treating physician before study enrolment. Required medication was ordered independently of the study.

### Study procedures and evaluations

After enrolment (V1), study data were collected during two consecutive treatment visits [i.e. week 4 ± 2 (V2) and week 12 ± 2 (V3)] and during the early close-out visit or the end of study (EOS) visit 24 weeks after study enrolment. During the first visit, the treating physicians recorded the patient’s demographic data (age, weight and sex), compliance with the inclusion and exclusion criteria, disease activity data on RA [DAS28; optionally, clinical disease activity index (CDAI)], data on prior (MTX and biologic DMARD) and concomitant (number and dosage of conventional synthetic DMARDs and/or CSs) pharmacological therapy, and relevant laboratory parameters (haematological parameters, CRP, ESR, liver enzymes, total cholesterol and triglyceride values). At all treatment visits (V1, V2, V3 and EOS), data related to DASs, relevant laboratory parameters and safety assessments were recorded. At the EOS visit (including early close-out visits), data on concomitant medical treatment (number and dosage of conventional synthetic DMARDs and/or CSs) were also recorded. The primary endpoint of the study was the change in either ESR- or CRP-based DAS28 count from baseline to week 24. Secondary objectives of the study were to determine the efficacy of treatment by CDAI and the number and percentage of patients achieving remission based on both DAS28 (≤2.6) and CDAI (≤2.8) from baseline to the end of study. Furthermore, efficacy was examined in subgroups defined based on prior RA treatment history: patients unsuccessfully treated with conventional synthetic DMARD therapy (1L), patients unsuccessfully treated with one biological medication (2L), and patients unsuccessfully treated with two or more biologicals (2L+). *Posthoc* subgroup analysis of the efficacy was performed by age (<45, 45–55, 55–65, 65–75 and >75 years), weight (<60, 60–75, 75–90 and >90 kg) and biological sex (male/female). During the study, the number of s.c. TCZ injections per patient, reason for MTX avoidance, prior treatment history, changes in the need for supplementary medications, including other DMARDs (SSZ, LEF, chloroquine, CsA, AZA and CYC) and CSs were monitored. CS tapering in patients with DAS28 remission was also evaluated.

### Safety measurements

All subjects who received at least one dose of treatment were included in the safety evaluation. Safety analysis covered the description of both non-serious and serious adverse events (AEs) or adverse events of special interest (AESIs), which were reported throughout the study and coded according to the actual version of the Medical Dictionary for Regulatory Activities (MedDRA, v24.1 September 2021). Additionally, an evaluation of adverse events according to incidence, intensity and relationship to therapy was performed.

### Statistical analysis

Analyses of demographic characteristics, laboratory parameters and efficacy endpoints were performed using descriptive statistical methods. Upon calculation of DAS28 scores, ESR and CRP values were used. If a patient had both values available, the ESR-based DAS28 value was included in the statistics, whereas the CRP-based DAS28 value was disregarded. The mean changes in DAS28 and CDAI described in the primary and secondary efficacy objectives were characterized by using point estimates and 95% CIs. Changes in efficacy parameters, between baseline and 24 weeks or between subgroups at a given study week, were analysed by Student’s paired or unpaired *t*-tests, respectively. A value of <0.05 was considered as significant. Statistical analyses were performed using SAS v.9.4 software (SAS Institute, Cary, NC, USA).

### Compliance with ethical standards

This study was carried out in the frame of the Declaration of Helsinki and in accordance with the rules of Good Pharmacoepidemiology Practices (ISPE/GPP). The study was approved by the National Institute of Pharmacy and Nutrition (OGYÉI, Hungary) and by the National Scientific and Ethical Committee of the Medical Research Council (ETT-TUKEB, Hungary).

## Results

**Fig. 1:**
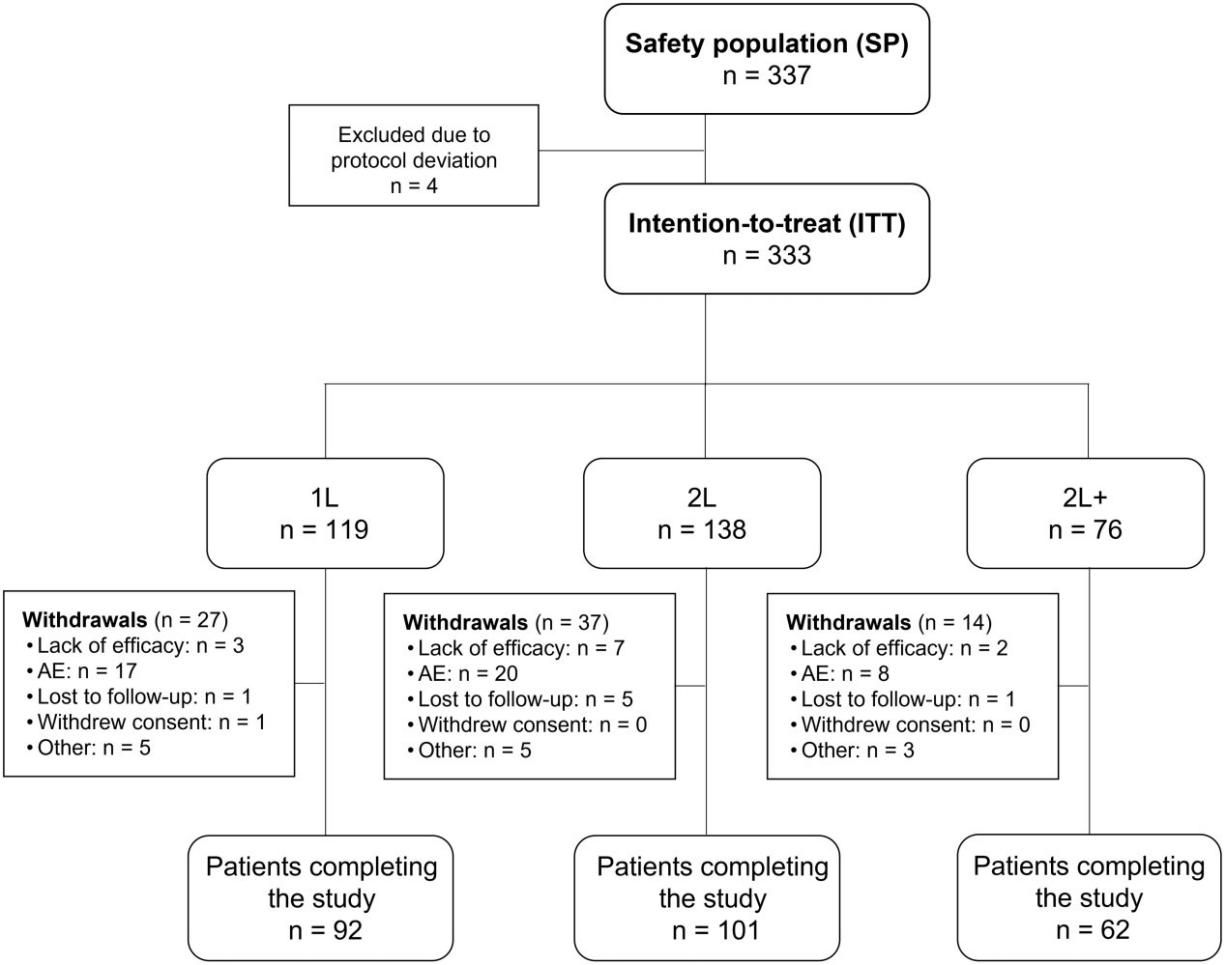
Study population flow chart AE: adverse event; *n*: number of patients in group. Subgroups: 1L: first line = tocilizumab after failing conventional synthetic DMARDs before enrolment; 2L: second line = tocilizumab after failing one biologic before enrolment; 2L+: third line = tocilizumab after failing two or more biologics before enrolment.

Between May 2015 and December 2018, a total number of 337 (50 male and 287 female) patients were enrolled at 18 centres in Hungary. During the data evaluation phase, the DAS28 values of four patients indicated remission at the time of enrolment, upon which the patients were excluded. Consequently, the intention-to-treat (ITT) population consisted of 333 patients. Demographic and safety analyses were performed on all included patients (Fig. 1).

The mean (s.d.) age of enrolled patients was 57.58 (11.78) years (range: 20.88–82.54 years), and their weight averaged (s.d.) at 72.8 (16.78) kg. The proportion of patients aged <65 years was higher (72.11%) compared with the proportion of patients aged ≥65 years (27.89%). Demographic data are presented in Table 1 and Supplementary Tables S1a and S1b (available at *Rheumatology Advances in Practice* online). From the 333 patients, 78 (23.42%) were withdrawn prematurely from the therapy, and 255 (76.58%) finished the 24-week treatment period as planned. Reasons for withdrawal were lack of efficacy (12 patients), adverse events (45 patients), lost to follow-up (7 patients), withdrawal of informed consent (1 patient) or other reasons (13 patients) (Fig. 1).

**Table 1:** Baseline patient demographics, disease characteristics and prior medical history

Parameter	Safety population (*n* = 337, unless stated otherwise)
**Demographics**	
Age, mean (s.d.), years	57.58 (11.78)
Weight, mean (s.d.), kg	72.8 (16.78)
Patient >65 years of age, *n* (%)	94 (27.89)
Male/female, *n* (%)	50/287 (14.84/85.16)
**Disease characteristics**	
DAS28 ESR at baseline, mean (s.d.) (*n* = 281)	5.79 (0.97)
DAS28 CRP at baseline, mean (s.d.) (*n* = 52)	5.8 (0.97)
CDAI at baseline, mean (s.d.) (*n* = 234)	32.36 (12.54)
**Treatment history before study enrolment or at baseline**	
MTX (*n* = 333)	
MTX stopped >1 week before TCZ start, *n* (%)	114 (34.23)
No MTX, *n* (%)	219 (65.77)
MTX dosage, mean (s.d.), mg/week (*n*=114)	15.48 (4.411)
Reason for no MTX use, *n* (%) (*n* = 219)	
Non-adherence	19 (8.68)
Intolerance or contraindications	195 (89.04)
Other	5 (2.28)
Conventional synthetic DMARDs (other than MTX) (*n* = 333)	
Conventional synthetic DMARD at enrolment, *n* (%)	185 (55.56)
Conventional synthetic DMARD continued after TCZ initiation, *n* (%)	119 (64.32)
Biologic treatment (*n* = 333)	
One biologic product (2L), *n* (%)	138 (41.44)
Two or more biologic products (2L+), *n* (%)	76 (22.82)
No biologic products (1L), *n* (%)	119 (35.74)
CSs (*n* = 333)	
CS treatment at baseline, *n* (%)	160 (48.05)
CS dosage at baseline, mean (s.d.), mg/month	171.6 (107.4)

CDAI: clinical disease activity index; *n*: number of patients in group; TCZ: tocilizumab. Defined subgroups: 1L: first line = tocilizumab after failing DMARDs before enrolment; 2L: second line = tocilizumab after failing one biologic before enrolment; 2L+: third line = tocilizumab after failing two or more biologics before enrolment.

Most of the subjects (323 patients, 95.85%) started the s.c. TCZ therapy at the time of the enrolment visit, whereas 14 patients (4.15%) were already under treatment before entering the study. The median number of injections was 24, ranging between 1 and 30 in the study population. Before s.c. TCZ therapy, 214 patients (64.26%) received one or more biologic products. From these, 138 received one and 76 patients received two or more biological treatments before study enrolment (Table 1 and Supplementary Table S2, available at *Rheumatology Advances in Practice* online).

**Fig. 2:**
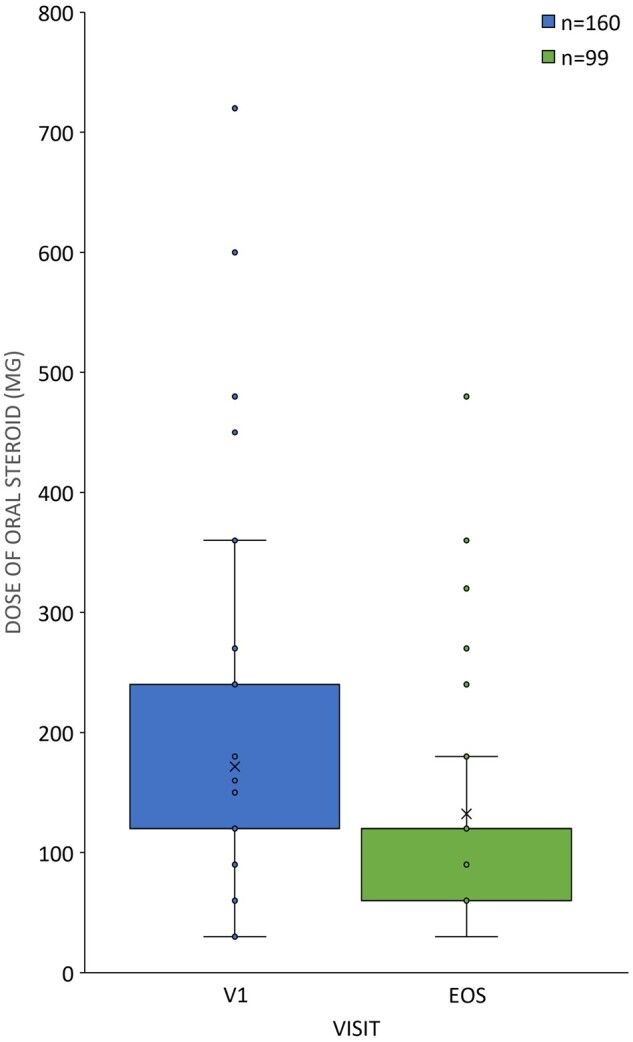
CS tapering in patients with DAS28 remission Dosages (in milligrams per month) of oral CS therapy in the intention-to-treat (ITT) population (*n* = 333) at the time of first tocilizumab therapy (V1: enrolment, *n* = 160, blue) *vs* the final visit (EOS: end of study, *n* = 99, green), plotted as box plots.

In total, 114 patients (34.23%) received MTX before the initiation of s.c. TCZ therapy at a mean (s.d.) dosage of 15.48 (4.411) mg/week (median: 15 mg/week, range: 5–25 mg/week). In their case, MTX therapy was terminated >1 week before the start of TCZ treatment. For the remaining 219 patients (65.77%), MTX treatment was not initiated in relationship to their current RA disease owing to previously established intolerance or non-adherence (Table 1). One hundred and eighty-five patients (55.56%) received conventional synthetic DMARD treatment (other than MTX) and 148 patients (44.44%) did not receive such treatment before the initiation of s.c. TCZ therapy. Other DMARD treatment was continued concomitantly for 119 patients (64.32%) after study enrolment (Table 1). The number of patients receiving supplementary DMARD decreased to 81 (24.32%) patients at the end of the 24-week treatment period. Oral CSs were administered to 160 patients (48.05%) at baseline, which decreased to 99 patients (29.73%) at the time of the final visit. The mean CS dosage was 171.6 (107.4) mg/month at the beginning of the study and decreased to 132.3 (83.5) mg/month at the time of the final visit (Fig. 2).

**Fig. 3:**
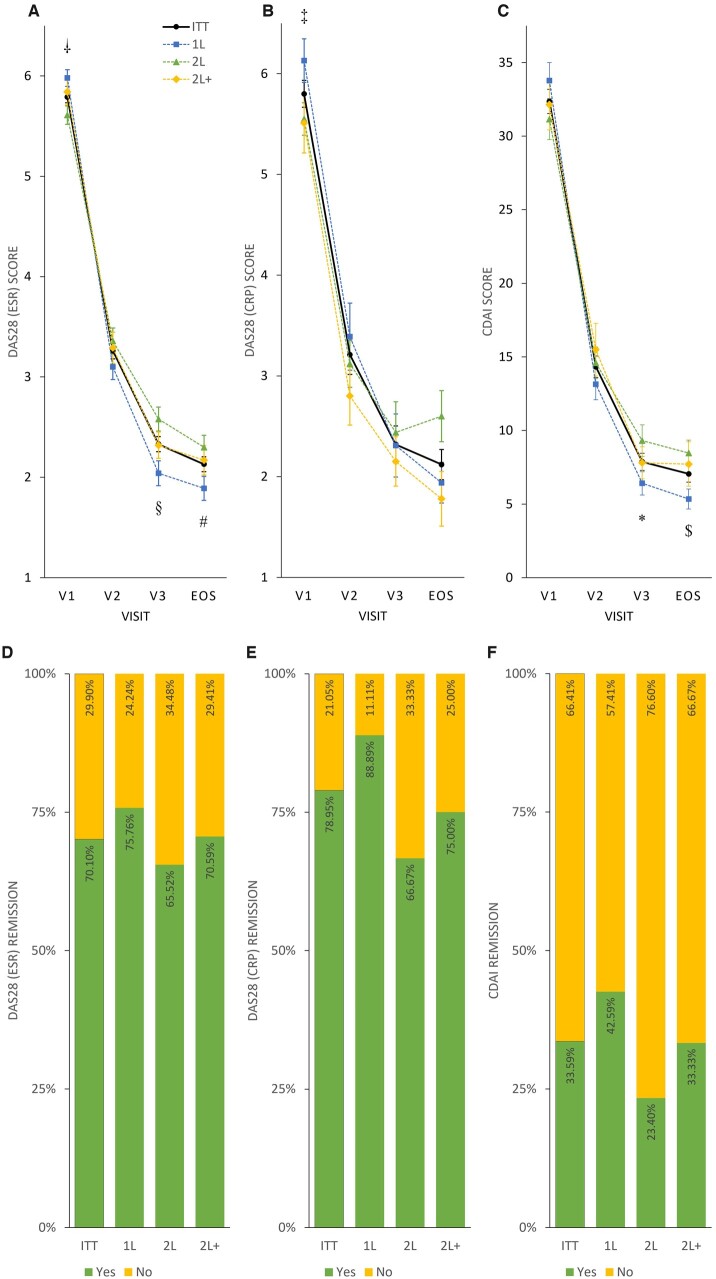
Disease activity and remission rates in the intention-to-treat and treatment subgroups Mean DAS28 ESR (**A**), DAS28 CRP (**B**) and CDAI (**C**) scores, respectively, plotted as line charts by visits (V1, V2, V3 and EOS). Symbols denote significant differences between subgroups: †*P* < 0.005, DAS28 ESR 1L *vs* 2L at V1 (1L > 2L); ^‡^*P* < 0.05, DAS28 CRP 1L *vs* 2L at V1 (1L > 2L); ^§^*P* < 0.005, DAS28 ESR 1L *vs* 2L at V3 (2L > 1L); *P < 0.05, CDAI 1L *vs* 2L at V3 (2L > 1L); ^#^*P* < 0.05, DAS28 ESR 1L *vs* 2L at EOS (2L > 1L); ^$^*P* < 0.01, CDAI 1L *vs* 2L at EOS (2L > 1L). Percentage of patients achieving remission (yes: green; no: yellow) evaluated by DAS28 ESR (**D**), DAS28 CRP (**E**) and CDAI (**F**) scores, plotted as stacked bar charts.

In total, 204 patients had ESR values and 19 patients had CRP values at both the enrolment and final visits. The mean (s.d.) decrease in DAS28 ESR and DAS28 CRP was 3.72 (1.365) and 3.64 (1.095) points, respectively. A significant difference from baseline to EOS in both cases was observed (*P *<* *0.0001). However, when interpreting the DAS28 CRP results, the small number of samples should be considered. In total, 128 patients had CDAI scores at both the enrolment and the final visits. A significant mean (s.d.) decrease in CDAI score 27.12 (13.633) was observed (*P *<* *0.0001) (Fig. 3A–C; Supplementary Table S3, available at *Rheumatology Advances in Practice* online).

In total, 70.10, 78.95 and 33.59% of the patients achieved remission if analysed by the changes in DAS28 ESR, DAS28 CRP and CDAI from baseline to EOS, respectively (Fig. 3D–F).

When considering DAS28 ESR, DAS28 CRP and CDAI values, significant differences between enrolment and final visits were observed for all subgroups (*P *<* *0.002 to *P *<* *0.0001; Supplementary Table S3, available at *Rheumatology Advances in Practice* online, Fig. 3A–C). A difference in baseline disease activity was apparent in 1L *vs* 2L subgroups when assessed using DAS28 ESR (*P *<* *0.005) or DAS28 CRP (*P *<* *0.05), indicating a higher RA activity in biologic treatment-naïve, 1L patients. Nonetheless, the response to TCZ treatment measured at the final visit was more pronounced in the 1L compared with the 2L subgroup (*P *<* *0.05 or *P *<* *0.01 assessed by DAS28 ESR or CDAI; Fig. 3A and C; Supplementary Table S3, available at *Rheumatology Advances in Practice* online).

DAS28 ESR and remission rates were 75.76, 65.52 and 70.59% and CRP remission rates 88.89, 66.67 and 75.00% for 1L, 2L and 2L+, respectively. CDAI remission rates were 42.59, 23.40 and 33.33% for 1L, 2L and 2L+, respectively (Fig. 3D–F).

**Fig. 4.**
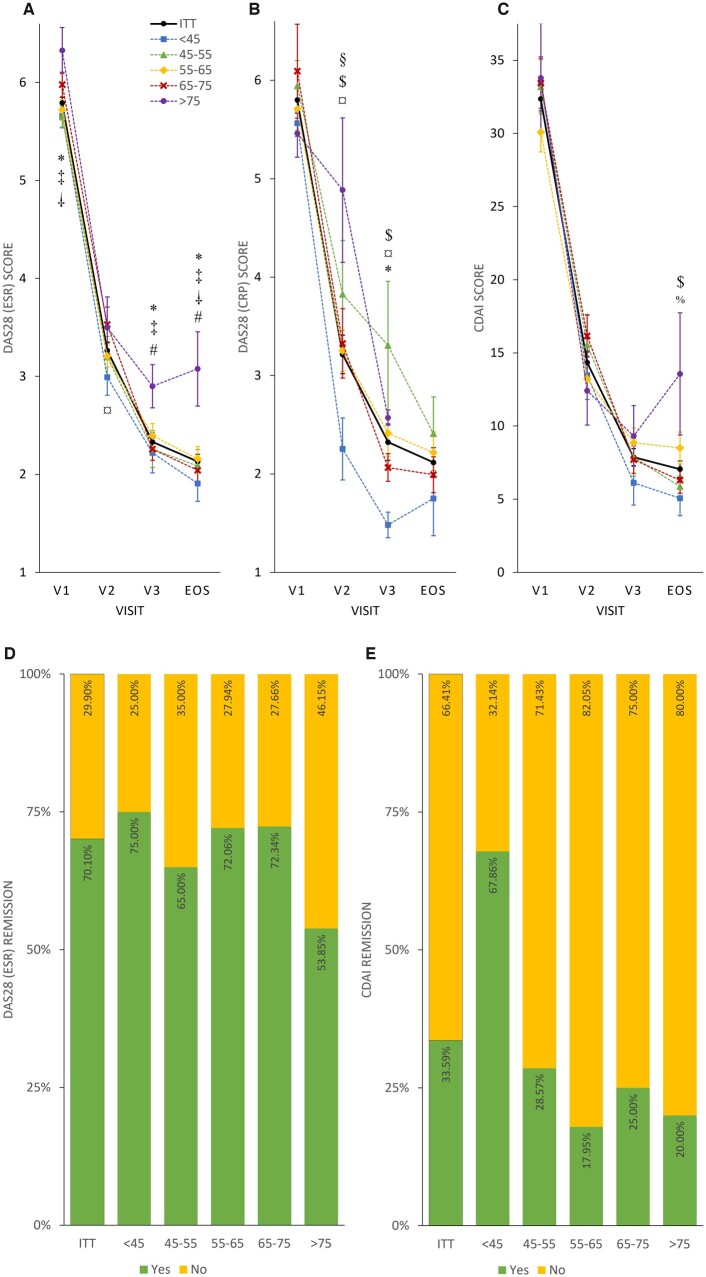
Disease activity and remission rate in age subgroups Mean DAS28 ESR (**A**), DAS28 CRP (**B**) and CDAI (**C**) scores plotted as line charts by visits (V1, V2, V3 and EOS). Symbols denote significant differences between subgroups: *P < 0.05 at the DAS28 ESR as ‘<45’ < ‘>75’ at V1; ^‡^*P* < 0.02 at the DAS28 ESR as ‘45–55’ < ‘>75’ at V1; ^†^*P* < 0.05 at the DAS28 ESR as ‘55–65’ < ‘>75’ at V1; ^¤^*P* < 0.05 at the DAS28 ESR as ‘<45’ < ‘65–75’ at V2; ^*^P < 0.05 at the DAS28 ESR as ‘<45’ < ‘>75’ at V3; ^‡^*P* < 0.05 at the DAS28 ESR as ‘45-55’ < ‘>75’ at V3; ^#^*P* < 0.05 at the DAS28 ESR as ‘65–75’ < ‘>75’ at V3; ^*^P < 0.02 at the DAS28 ESR as ‘<45’ < ‘>75’ at EOS; ^‡^*P* < 0.05 at the DAS28 ESR as ‘45–55’ < ‘>75’ at EOS; ^†^*P* < 0.05 at the DAS28 ESR as ‘55–65’ < ‘>75’ at EOS; ^#^*P* < 0.05 at the DAS28 ESR as ‘65–75’ < ‘>75’ at EOS; ^§^*P* < 0.05 at the DAS28 CRP as ‘<45’ < ‘45–55’ at V2; ^$^*P* < 0.05 at the DAS28 CRP as ‘<45’ < ‘55–65’ at V2; ^¤^*P* < 0.05 at the DAS28 CRP as ‘<45’ < ‘65–75’ at V2; ^$^*P* < 0.005 at the DAS28 CRP as ‘<45’ < ‘55–65’ at V3; ^¤^*P* < 0.05 at the DAS28 CRP as ‘<45’ < ‘65–75’ at V3; ^*^P < 0.002 at the DAS28 CRP as ‘<45’ < ‘>75’ at V3; ^$^*P* < 0.05 at the CDAI as ‘<45’ < ‘55–65’ at EOS; ^%^*P* < 0.05 at the CDAI as ‘45–55’ < ‘55–65’ at EOS. Percentage of patients achieving remission (yes: green; no: yellow) in age groups evaluated by DAS28 ESR (**D**) and CDAI (E) scores, presented as stacked bar charts. Sample size was insufficient for DAS28 CRP age subgroup assessment.

*Post* *hoc* subgroup analysis revealed that after 24 weeks patients aged >75 years were more likely to have smaller differences in DAS28 ESR or CDAI values and less often reached remission compared with younger patients (Fig. 4; Supplementary Table S4a, available at *Rheumatology Advances in Practice* online, for more details). Younger age was more likely to result in better disease control; patients <45 years reached CDAI remission two times more frequently than the whole ITT population (67.86 *vs* 33.59%; Fig. 4D–F). Weight or sex was not associated with clinical response to TCZ at EOS; however, patients with a lower body weight responded transiently to TCZ at V3 to a lesser extent regarding DAS28 ESR or CDAI values. No correlation between age and weight was revealed (Pearson’s correlation coefficient: −0.0072; Supplementary Figs S1–S3 and Supplementary Tables S5a and S6a, available at *Rheumatology Advances in Practice* online).

A total of 145 AEs were reported by 117 (34.72%) patients (Table 2), most of which were categorized as mild to moderate. Seven AEs manifested in six patients (1.78%) were considered severe. Fifty-seven patients had AEs (16.91%) that were considered to be related to the study drug. Twelve patients (3.56%) reported 13 serious AEs, and 21 patients (6.23%) reported 21 AESIs. Treatment with s.c. TCZ was discontinued in 46 patients (13.65%) as a result of AEs. The most common AEs by MedDRA System Organ Class (SOC) were ‘investigations’ in 31 patients (9.20%) followed by ‘infections and infestations’ in 30 patients (8.90%) and ‘general disorders and administration site conditions’, which were described in 20 patients (5.93%). The most common AE by preferred term was ineffectiveness of the drug, which was reported by 14 (9.66%) patients. A total of 11 patients (3.26%) experienced hypersensitivity, and 11 (3.26%) patients had decreased neutrophil count; none of which was classified as severe. No pregnancies or any AEs related to overdose were reported during the study.

**Table 2:** Adverse events reported during the study

Adverse event	Safety population [*n* = 337]
Total number of AEs	117 (34.72) [145]
Serious AEs	12 (3.56) [13]
AESIs	21 (6.23) [21]
Patient discontinuation owing to AE	62 (18.40) [72]
Treatment-related AE	57 (17.91) [64]
Severe AE	6 (1.78) [7]
Most common AE by SOC	
Blood and lymphatic system disorders^a^	10 (2.97) [10]
Leucopenia^b^	2 (0.59) [2]
Neutropenia	3 (0.89) [3]
Thrombocytopenia	4 (1.19) [4]
Gastrointestinal disorders	6 (1.78) [7]
Diarrhoea	3 (0.89) [3]
General disorders and administration site conditions	20 (5.93) [22]
Drug ineffective	14 (4.15) [14]
Peripheral oedema	2 (0.59) [2]
Immune system disorders	11 (3.26) [11]
Hypersensitivity	11 (3.26) [11]
Infections and infestations	30 (8.90) [32]
Bronchitis	2 (0.59) [3]
Erysipelas	2 (0.59) [2]
Nasopharyngitis	2 (0.59) [2]
Pneumonia	5 (1.48) [5]
Upper respiratory tract infection	3 (0.89) [3]
Urinary tract infection	2 (0.59) [2]
Injury, poisoning and procedural complications	5 (1.48) [5]
Investigations	31 (9.20) [36]
Alanine aminotransferase increased	4 (1.19) [4]
γ-Glutamyl transferase increased	4 (1.19) [4]
Hepatic enzyme increased	8 (2.37) [8]
Neutrophil count decreased	11 (3.26) [11]
White blood cell count decreased	4 (1.19) [4]
Metabolism and nutrition disorders	2 (0.59) [2]
Neoplasms benign, malignant and unspecified (including cysts and polyps)	2 (0.59) [2]
Skin and subcutaneous tissue disorders	10 (6.90) [10]
Rash	3 (0.89) [3]
Vascular disorders	4 (1.19) [4]

All values are reported as *n* = number of patients, incidence in the safety population (SP, %) and [number of individual occurrences].

a The most common AEs are reported for system organ classes occurring in ≥0.5% of patients.

b The most common AEs are reported by preferred term in ≥0.5% of patients.

AE: adverse event; AESI: adverse event of special interest; SOC: system organ class.

Reported infections included pneumonia, upper respiratory tract infection, bronchitis, erysipelas, nasopharyngitis and urinary tract infection, endocarditis, fungal infection, Herpes zoster, infection, influenza, lung infection, mastoiditis, muscle abscess, pharyngitis, pyoderma, pustular rash, respiratory tract infection, rhinitis, sinusitis and viral respiratory tract infection. Laboratory findings indicated increased liver enzymes, cholesterol, alanine aminotransferase, aspartate aminotransferase, γ-glutamyl transferase or lymphocyte count, or decreased neutrophil, platelet or white blood cell count. Rash was most frequently reported as skin and connective tissue-related disorder. Breast cancer, the only malignancy reported during the study, in one case was judged to be unrelated to the TCZ treatment. The serious AEs reported were pneumonia, breast cancer, dyspnoea, endocarditis, gastric perforation, haematochezia, hyponatraemia, infection of unknown origin, mastoiditis, muscle abscess, pyoderma and thrombophlebitis. The most frequently reported AESI was hypersensitivity (3.26%), which led to treatment discontinuation in all cases. No deaths were reported during the study (Table 2).

## Discussion

The present real-world study enrolled patients for whom MTX was contraindicated, whereas concomitant treatment with other conventional synthetic DMARDS, CSs or NSAIDs was allowed. Subcutaneous TCZ treatment for 24 weeks resulted in improved efficacy parameters, including DAS28 ESR (average decrease −3.72 ± 1.365), DAS28 CRP (average decrease 3.64 ± 1.095) and CDAI (average decrease 27.12 ± 13.633) in the ITT population. These observations are in line with other recent real-world studies of TCZ administered s.c. [22, 23]; except for a considerably lower-grade change in CDAI (−18.29 ± 14.52) reported in Israel [22]. As in other clinical studies [13, 24], changes in DAS28 and CDAI scores were significantly higher in patients receiving s.c. TCZ as a first-line biologic treatment compared with patients pretreated with biologic agents before study enrolment.

At 24 weeks, 70.10% of the ITT population had DAS28 ESR remission. This observation is similar to other recent real-world studies of s.c. TCZ treatment (75.4% in ACT-MOVE [25] or 59.5% in ML28700 [23]). Although TCZ was administered for 52 weeks in the ACT-MOVE study, the DAS28 scores stabilized around 24 weeks, with a relatively small further increase in the remission rates [25], a phenomenon also reported in multiple RCTs in which patients receiving TCZ treatment were followed for an extended period of time [16, 23, 26–28]. However, in the phase 3 RCTs BREVACTA, SUMMACTA or MUSHASHI, considerably lower DAS28 remission rates were reported (32–49.7%) [15, 29, 30].

Patients without previous biologic treatment achieved remission according to DAS28 ESR or CRP in a higher proportion (75.76 or 88.89%, respectively) compared with those treated with one (65.52 or 66.67%) or more biologics (70.59 or 75.00%) before the s.c. TCZ therapy. It is noteworthy that EULAR definition and guidance were recently provided to support the management of difficult-to-treat RA patients, a subpopulation of patients who failed at least two biologic DMARDs [31, 32]. Likewise, the rates of patients with low disease activity or remission based on DAS28 and CDAI were consistently higher among TNF inhibitor-naïve patients compared with patients previously or recently treated with TNF inhibitor in the ACT-SURE and ROUTINE studies [13, 24].

The proportion of patients reaching CDAI remission (33.59%) was similar to that reported in the ACT-MOVE real-life [25] or TOZURA phase 4 [33] studies. Somewhat lower CDAI remission rates (∼16.5%) were reported in the MUSHASHI and a real-world study conducted in Israel [15, 22]. Unlike DAS28 scores, CDAI does not include ESR or CRP levels, which could mitigate potential overestimation of remission rates.

The present study suggests an age-related decrease in efficacy of TCZ therapy. Compared with younger patients, TCZ therapy seemed to be less effective in patients >75 years of age, as evidenced by a decreased change in DASs and a lower remission rate compared with the whole study population. In parallel, a >2-fold increase in CDAI remission rates was observed in patients <45 years of age. Although real-world clinical data on TCZ therapy in elderly patients is limited, the REAC-TION study proposed that younger age was associated with a better clinical response and remission rate at 6 months after TCZ initiation [34]. Also, our findings are in line with recent reports in which registry data were used to analyse the clinical efficacy of TCZ in elderly patients [35, 36].

Along with previous studies and reviews, weight (expressed as BMI) [37, 38] and gender [39] were not associated with clinical response to TCZ among RA patients.

In the majority of RA patients, oral glucocorticoid therapy is initiated at the same time that DMARD therapy is initiated [33]. In general, RA clinical trials do not exclude these patients from participation if they are receiving a stable CS dosage at baseline, and they often compose 40–60% of the study population. However, CS administration should be tapered as soon as clinically feasible owing to the well-established risks of its own side effects [8]. In the SIMPACT study, 48.05% of patients received oral CSs at baseline at a mean dosage of 170.6 mg/month. These initial values could be decreased to 29.73% and 132.3 mg/month without worsening disease activity. This is in line with the results of an RCT in which effective disease control was achieved both with or without CS administration, indicating that CS use did not impact the efficacy of s.c. TCZ either as monotherapy or in combination with concomitant conventional synthetic DMARDs [33].

An inherent limitation of non-interventional, observational studies is the risk of selection bias. Moreover, missing observations owing to study drop-outs over time might distort study results. During the SIMPACT study, 23.44% of all initially enrolled patients discontinued the study. Although this drop-out percentage is higher than that reported in RCTs with s.c. TCZ administration (ranging from 7 to 14%), it is, however, comparable to previous real-world studies (ranging from 15 to 22%) [15, 29, 30]. In general, the AE profile of s.c. TCZ in this study was consistent with previous data. However, when comparing the safety data with previous RCTs or real-world studies, AEs might have been under-reported here, also a known phenomenon in real-world studies. For example, 34.72% of all patients experienced at least one AE in the SIMPACT study, whereas it ranged between 62.5 and 97.5% in previous real-world studies or controlled clinical trials with s.c. TCZ treatment [15, 16, 22, 23, 25, 29, 30, 33, 40]. Of note, a previous real-world study performed in Japan including 783 TCZ-naïve RA patients reported a similar AE proportion of 29.5% compared with the SIMPACT study [41].

In the SIMPACT study, 3.56% of all patients reported serious AEs, which is in line with previous data [15, 16, 22, 23, 25, 29, 30, 33, 40, 41]. Also, 6.23% of all patients experienced AESIs, which is similar to the 8.8% previously reported in a real-world study examining TCZ administered s.c. [23]. Compared with previous reports, the SIMPACT study had a similar AE profile. The most common AEs were changes in liver enzymes (4.75%) and infections and infestations (8.9%). Besides 2.1% of all patients who were diagnosed with severe infections (pneumonia and erysipelas), uncomplicated infections of the upper airways were most commonly reported. Additionally, the SIMPACT study lacked a control arm and was subject to limitations generally associated with real-world studies, such as inclusion and expectation bias. These limitations, however, are offset, at least in part, by the benefits of including a broader, less selected patient population.

A novel aspect of our study compared with the other published observational clinical studies performed with s.c. TCZ is the exclusive enrolment of patients for whom MTX was contraindicated. Previous studies indicated that s.c. TCZ monotherapy is non-inferior to s.c. TCZ plus MTX treatment, both in RCTs [3] and in standard clinical practice [25]. In the SIMPACT study, 44.44% of the study population received s.c. TCZ as monotherapy, while the remainder of the study population received concomitant conventional synthetic DMARDs after study enrolment. In the present study, no efficacy and safety analyses were performed on these groups separately. Although the small number of patients (*n* = 21) enrolled into the s.c. TCZ monotherapy arm of the ACT-MOVE study limited the conclusions that could be drawn specifically for this group in a real-world setting, the reported data regarding efficacy and safety of the monotherapy arm were similar to those of the SIMPACT study [3].

In conclusion, the SIMPACT study provides evidence that the efficacy and safety of s.c. TCZ in the real world are similar to observations during clinical development, even in the absence of MTX. Thus, the data collected in the SIMPACT study indicate that s.c. TCZ is effective when administered in a usual care setting and has a tolerability profile as previously established in RCTs.

## Supplementary Material

rkac038_Supplementary_Data
