# Supplementary material for: Real-world evidence on methotrexate-free subcutaneous tocilizumab therapy in patients with rheumatoid arthritis: 24-week data from the SIMPACT study
Source: Rheumatol Adv Pract. 2022 May 16;6(2):rkac038. doi: 10.1093/rap/rkac038 (PMC9154320; doi:10.1093/rap/rkac038)
Supplement: rkac038_Supplementary_Data [file rkac038_supplementary_data.docx]

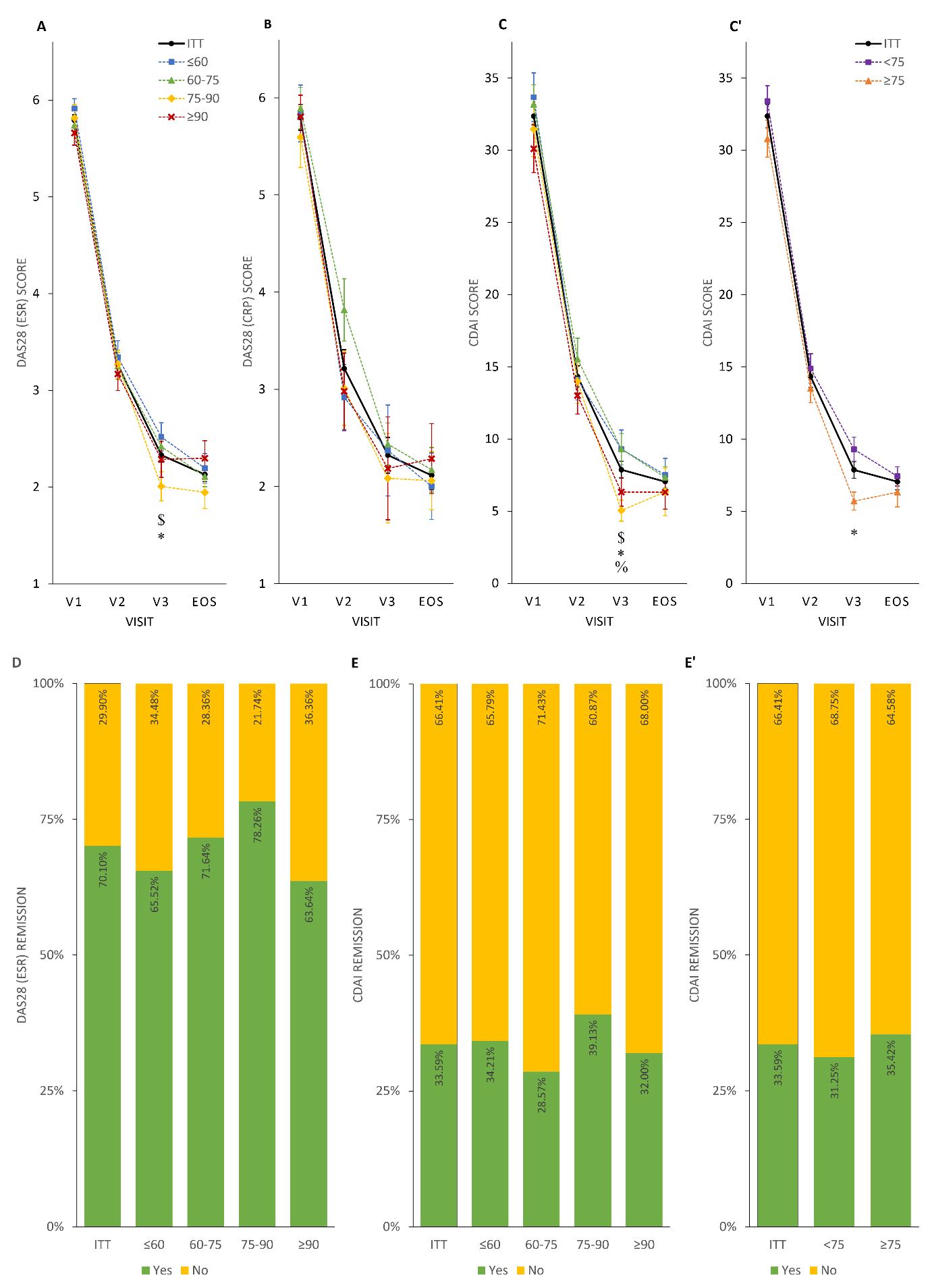


**Supplementary Figure S1. Disease activity in body weight subgroups.** First row: changes in Disease Activity Score-28 (DAS28) and Clinical Disease Activity Index (CDAI) scores evaluated by visits. Mean DAS28 ESR (erythrocyte sedimentation rate based DAS28; panel A), DAS28 CRP (C-reactive protein based DAS28; panel B), and CDAI (panels C and C’) scores, respectively, with standard error bars, plotted as line charts by visits (V1: enrolment, V2: week 4 ± 2, V3: week 12 ± 2, EOS: End of Study). Intention-to-treat population (ITT; n = 333) (black) includes all body weight subgroups [panels A, B, C: ≤60 = less than or equal to 60 kg (blue), 60-75 = greater than 60 kg but less than 75 kg (green), 75-90 = equal to or greater than 75 kg but less than 90 kg (yellow), ≥90 = equal to or greater than 90 kg (red); panel C’: <75 = less than 75kg (purple), ≥75 = equal to or greater than 75 kg (orange); all at the time of V1], that are superimposed, respectively. Significant differences between body weight subgroups were noted as follows: ^$^*p* < 0.02 at the DAS28 ESR as ‘≤60’ > ’75-90’ at V3; **p* < 0.05 at the DAS28 ESR as ’60-75’ > ’75-90’ at V3; ^$^*p* < 0.01 at the CDAI as ‘≤60’ > ’75-90’ at V3; **p* < 0.002 at the CDAI as ’60-75’ > ’75-90’ at V3; ^%^*p* < 0.05 at the CDAI as ’60-75’ > ’ ≥90’ at V3; **p* < 0.001 at the CDAI as ’<75’ > ’ ≥75’ at V3; *p* values are results of unpaired t-tests. Second row: remission rates in the ITT population (bars with solid black borders) and in the body weight subgroups (panels D, E: ≤60, 60-75, 75-90, ≥90; panel E’: <75, ≥75; borderless bars). Percentage of patients achieving remission (Yes: green, No: yellow) evaluated by DAS28 ESR (panel D), and CDAI (panels E and E’) scores, respectively, presented as stacked bar charts. Sample size was insufficient for DAS28 CRP body weight subgroup remission rate assessment.


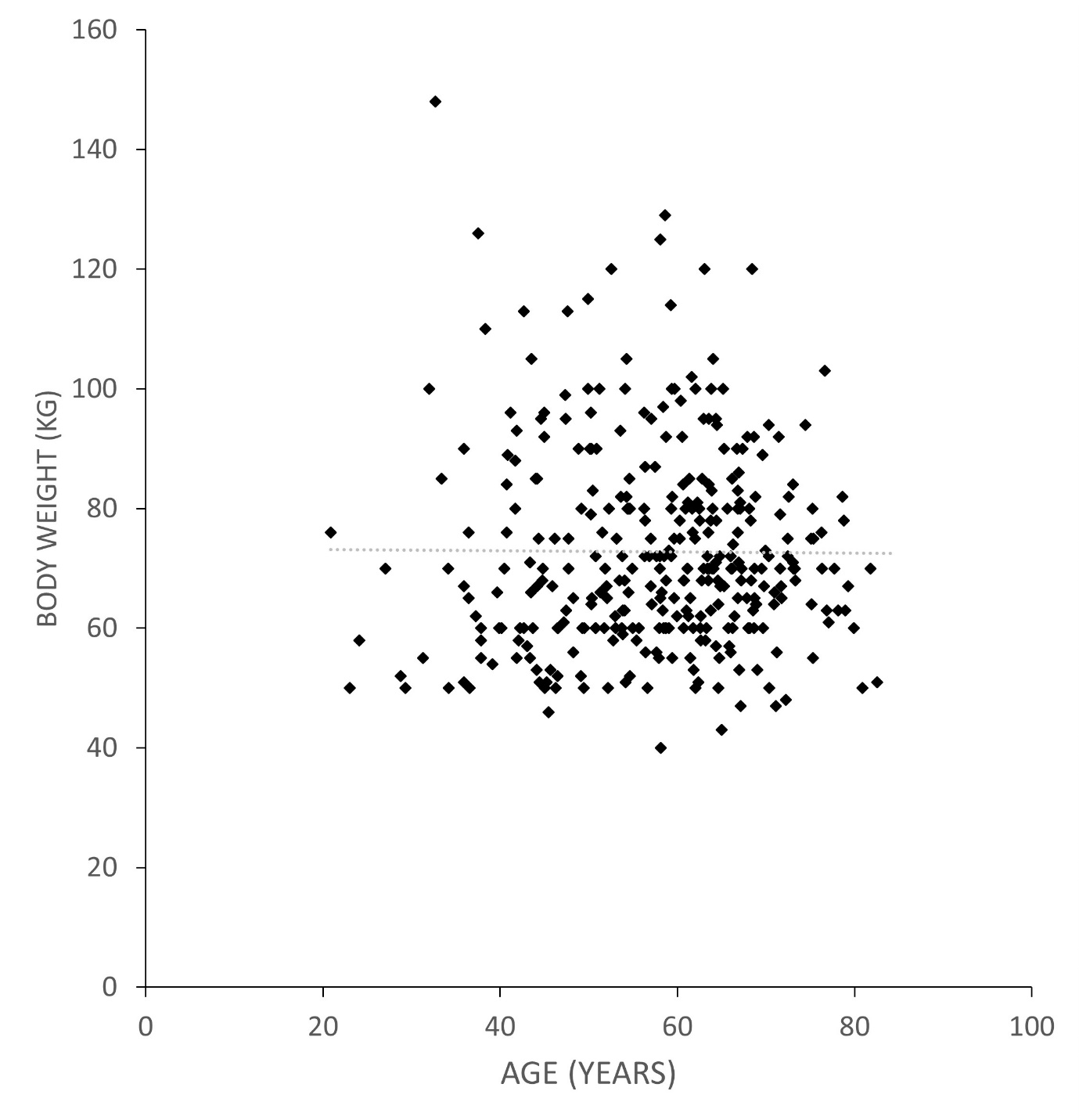


**Supplementary Figure S2. Correlation analysis between the age and body weight variables in the ITT population.** The age (x-axis, years) and body weight (y-axis, kg) variables of the ITT (n = 333) population plotted as a scatter chart. The calculated Pearson's correlation coefficient equals to -0.0072.


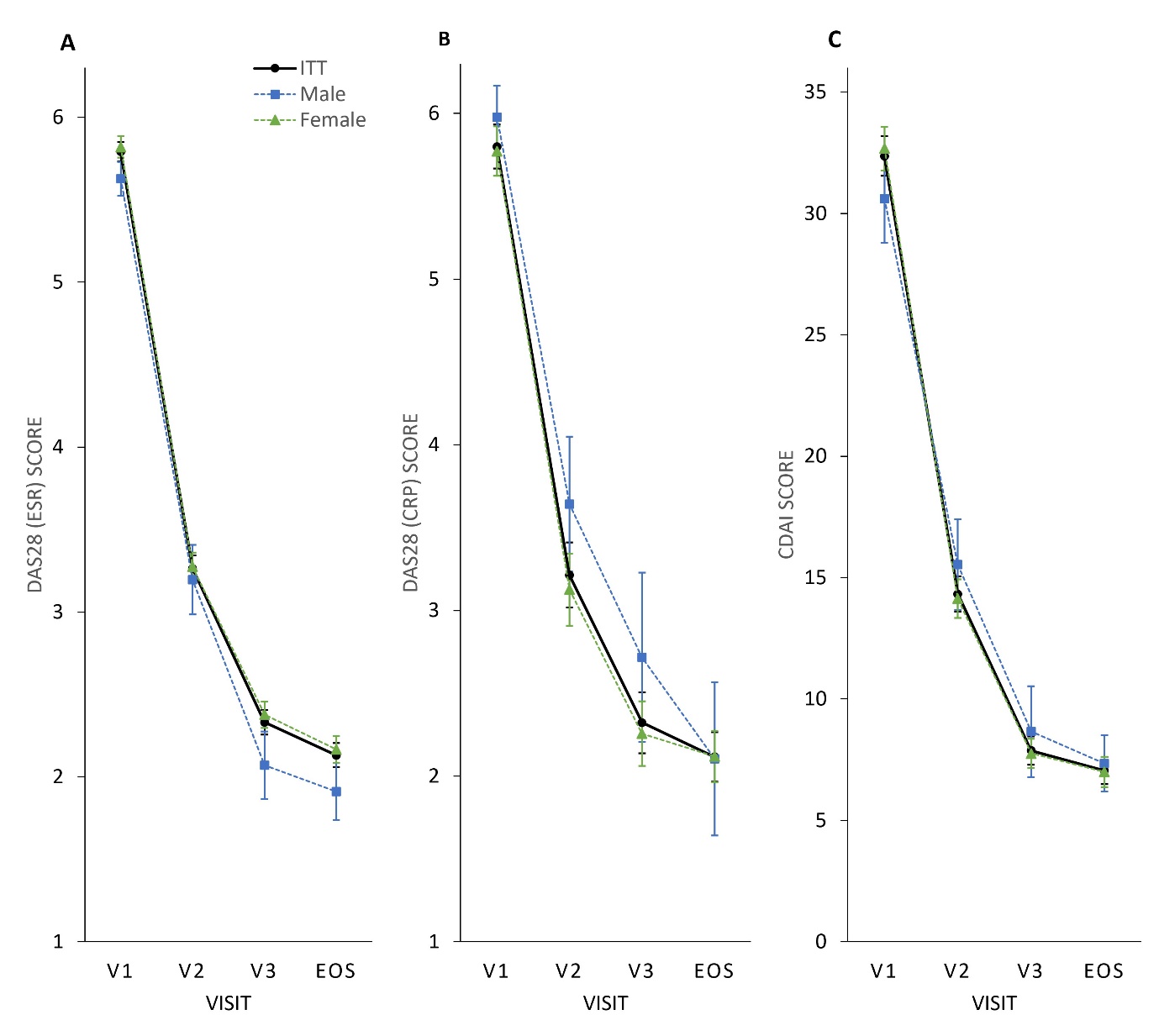


**Supplementary Figure S3. Disease activity in gender subgroups.** Changes in Disease Activity Score-28 (DAS28) and Clinical Disease Activity Index (CDAI) scores evaluated by visits. Mean DAS28 ESR (erythrocyte sedimentation rate based DAS28; panel A), DAS28 CRP (C-reactive protein based DAS28; panel B), and CDAI (panel C) scores, respectively, with standard error bars, plotted as line charts by visits (V1: enrolment, V2: week 4 ± 2, V3: week 12 ± 2, EOS: End of Study). Intention-to-treat population (ITT; n = 333) (black) includes both gender subgroups: male (blue)/female (green), that are superimposed, respectively. No significant differences were observed between the gender subgroups.

**Supplementary Table S1a. Demographic characteristics of patients in the safety population and subgroups defined according to prior RA treatment history.**

|  | **Safety population (SP)**  [n = 337] | **1L**  [n = 120] | **2L**  [n = 141] | **2L+**  [n = 76] |
| --- | --- | --- | --- | --- |
| Age (years); mean (SD) | 57.58 (11.78) | 57.06 (11.87) | 57.41 (11.78) | 58.74 (11.71) |
| Weight (kg); mean (SD) | 72.8 (16.78) | 72.90 (18.45) | 73.06 (15.51) | 72.17 (16.48) |
| Patient > 65 years; n (%) | 94 (27.89) | 35 (29.17) | 37 (26.24) | 22 (28.95) |
| Male / Female; n (%) | 50 / 287 (14.84 / 85.16) | 22 / 98 (18.33 / 81.67) | 24 / 117 (17.02 / 82.98) | 4 / 72 (5.26 / 94.74) |

n = number of patients in group; SD = Standard deviation; SP = safety population; Defined subgroups: 1L = first line: tocilizumab after failing DMARDs, 2L = second line: tocilizumab after failing one biological, 2L+ = third line: tocilizumab after failing two or more biologicals – prior to enrolment, respectively.

**Supplementary Table S1b. Demographic characteristics of patients in the intent-to-treat population and subgroups defined according to prior RA treatment history.**

|  | **Intention-to-treat (ITT) population**  [n = 333] | **1L**  [n = 119] | **2L**  [n = 138] | **2L+**  [n = 76] |
| --- | --- | --- | --- | --- |
| Age (years); mean (SD) | 57.51 (11.80) | 57.08 (11.92) | 57.19 (11.80) | 58.74 (11.71) |
| Weight (kg); mean (SD) | 72.73 (16.76) | 72.63 (18.29) | 73.12 (15.61) | 72.17 (16.48) |
| Patient > 65 years; n (%) | 92 (27.63) | 35 (29.41) | 35 (25.36) | 22 (28.95) |
| Male / Female; n (%) | 49 / 284 (14.71 / 85.29) | 21 / 98 (17.65 / 82.35) | 24 / 114 (17.39 / 82.61) | 4 / 72 (5.26 / 94.74) |

n = number of patients in group; SD = Standard deviation; ITT = intent-to-treat population; Defined subgroups: 1L = first line: tocilizumab after failing DMARDs, 2L = second line: tocilizumab after failing one biological, 2L+ = third line: tocilizumab after failing two or more biologicals – prior to enrolment, respectively.

**Supplementary Table S2. Distribution and frequency of biologicals prior to subcutaneous TCZ treatment.**

| **Name of biological product** | **n** | **%** |
| --- | --- | --- |
| Adalimumab   - *adalimumab only* - *adalimumab + other biological* | 102  *44*  *58* | 47.00  *20.27*  *26.73* |
| Etanercept   - *etanercept only* - *etanercept + other biological* | 73  *32*  *41* | 33.64  *14.75*  *18.89* |
| Infliximab   - *infliximab only* - *infliximab + other biological* | 9  *4*  *5* | 4.15  *1.85*  *2.30* |
| Golimumab   - *golimumab only* - *golimumab + other biological* | 30  *15*  *15* | 13.82  *6.91*  *6.91* |
| Certolizumab pegol   - *certolizumab pegol only* - *certolizumab pegol + other biological* | 66  *31*  *35* | 30.41  *14.28*  *16.13* |
| Abatacept   - *abatacept only* - *abatacept + other biological* | 21  *10*  *11* | 9.68  *4.61*  *5.07* |
| Rituximab   - *rituximab only* - *rituximab + other biological* | 12  *1*  *11* | 5.53  *0.46*  *5.07* |
| Other   - *RA0077 clinical study* - *biosimilar rituximab* - *leflunomide +tauredon* - *IL-6 receptor blocker* - *clazakizumab* - *ustekinumab* - *ustekinumab +Janus (JAK) kinase inhibitor* - *sekukinumab* - *biosimilar etanercept* | 9  *1*  *1*  *1*  *1*  *1*  *1*  *1*  *1*  *1* | 4.15  *0.46*  *0.46*  *0.46*  *0.46*  *0.46*  *0.46*  *0.46*  *0.46*  *0.46* |

n = number of patients receiving biological therapy.

**Supplementary Table S3. Change in disease activity parameters from baseline to the end of study.**

| ***Population*** | ***Disease activity parameter*** | ***DAS28 ESR*** | | | ***DAS28 CRP*** | | | ***CDAI*** | | |
| --- | --- | --- | --- | --- | --- | --- | --- | --- | --- | --- |
|  |  | **V1** | **EOS** | **Change** | **V1** | **EOS** | **Change** | **V1** | **EOS** | **Change** |
| *ITT*  *[n = 333]* | n value | 281 | 225 | 204 | 52 | 32 | 19 | 234 | 156 | 128 |
|  | Mean (SD) | 5.79 (0.968) | 2.13 (1.111) | -3.72 (1.365)* | 5.8 (0.965) | 2.12 (0.864) | -3.64 (1.095)* | 32.36 (12.540) | 7.05 (7.014) | -27.12 (13.633)* |
|  | 95%CI | (5.68; 5.90) | (1.98; 2.28) | (-3.91; -3.53) | (5.53; 6.07) | (1.8; 2.43) | (-4.17; -3.11) | (30.75; 33.98) | (5.94; 8.16) | (-29.50; -24.73) |
| *1L*  *[n = 119]* | n value | 96 | 76 | 66 | 23 | 16 | 9 | 90 | 62 | 54 |
|  | Mean (SD) | 5.98 (0.816) | 1.89 (1.045) | -4.13 (1.295)* | 6.13 (1.056) | 1.94 (0.835) | -4.09 (0.739)* | 33.77 (11.574) | 5.34 (5.343) | -29.55 (12.712)* |
|  | 95%CI | (5.81; 6.14) | (1.65; 2.13) | (-4.45; -3.81) | (5.68; 6.59) | (1.49; 2.38) | (-4.66; -3.53) | (31.35; 36.20) | (3.99; 6.70) | (-33.02; -26.08) |
| *2L*  *[n = 138]* | n value | 119 | 93 | 87 | 19 | 10 | 6 | 96 | 57 | 47 |
|  | Mean (SD) | 5.61 (1.012) | 2.3 (1.143) | -3.41 (1.387)* | 5.55 (0.725) | 2.6 (0.846) | -2.85 (0.871) | 31.14 (13.562) | 8.47 (6.612) | -23.39 (15.034)* |
|  | 95%CI | (5.43; 5.8) | (2.06; 2.53) | (-3.7; -3.11) | (5.2; 5.9) | (2.00; 3.21) | (-3.77; -1.94)^$^ | (28.40; 33.89) | (6.71; 10.22) | (-27.80; -18.97) |
| *2L+*  *[n = 76]* | n value | 66 | 56 | 51 | 10 | 6 | 4 | 48 | 37 | 27 |
|  | Mean (SD) | 5.84 (1.044) | 2.17 (1.108) | -3.73 (1.297)* | 5.51 (0.995) | 1.78 (0.726) | -3.79 (1.606) ^#^ | 32.14 (11.866) | 7.72 (9.155) | -28.74 (11.216)* |
|  | 95%CI | (5.58; 6.1) | (1.88; 2.47) | (-4.0; -3.37) | (4.8; 6.23) | (1.02; 2.54) | (-6.35; -1.24) | (28.70; 35.59) | (4.67; 10.77) | (-33.18; -24.31) |

SD = standard deviation; CI = confidence interval; V1 = first visit, baseline; EOS = end of study visit; n = number of patients in group; n value = number of patients with Disease Activity Score-28 (DAS28) or Clinical Disease Activity Index (CDAI) values at baseline or end of study; ITT = intent-to-treat population; ESR = erythrocyte sedimentation rate; CRP = C-reactive protein; DMARD = disease-modifying anti-rheumatic drugs. Defined subgroups: 1L = first line: tocilizumab after failing DMARDs, 2L = second line: tocilizumab after failing one biological, 2L+ = third line: tocilizumab after failing two or more biologicals – prior to enrolment, respectively. **p* = 0.0001; ^$^*p* = 0.0005; ^#^*p* = 0.0180 baseline vs. end of study values, using paired t-test.

**Supplementary Table S4a. Unpaired t-test results of all age subgroups at all visits.**

|  |  |  | **DAS28 ESR** | | | |  | **DAS28 CRP** | | | |  | **CDAI** | | | |
| --- | --- | --- | --- | --- | --- | --- | --- | --- | --- | --- | --- | --- | --- | --- | --- | --- |
|  |  |  |  |  |  |  |  |  |  |  |  |  |  |  |  |  |
| ***p*(T<=t) two-tail** |  |  | **V1** | **V2** | **V3** | **EOS** |  | **V1** | **V2** | **V3** | **EOS** |  | **V1** | **V2** | **V3** | **EOS** |
| **<45/45-55** | **§** |  | 0.9642 | 0.4578 | 0.8931 | 0.4773 |  | 0.2214 | **0.0388** | 0.0547 | 0.2786 |  | 0.9342 | 0.4461 | 0.3918 | 0.5765 |
| **<45/55-65** | **$** |  | 0.7038 | 0.3247 | 0.4729 | 0.2612 |  | 0.5889 | **0.0227** | **0.0037** | 0.3362 |  | 0.1540 | 0.8965 | 0.1436 | **0.0402** |
| **<45/65-75** | **¤** |  | 0.0705 | **0.0408** | 0.8772 | 0.5418 |  | 0.3475 | **0.0491** | **0.0298** | 0.6096 |  | 0.9889 | 0.2334 | 0.3857 | 0.4201 |
| **<45/>75** | ***** |  | **0.0209** | 0.1841 | **0.0366** | **0.0145** |  | 0.7728 | 0.2501 | **0.0016** | - |  | 0.9298 | 0.7309 | 0.2619 | 0.1036 |
| **45-55/55-65** | **%** |  | 0.6509 | 0.9770 | 0.5469 | 0.7271 |  | 0.5064 | 0.3801 | 0.2847 | 0.6892 |  | 0.1829 | 0.3228 | 0.5535 | **0.0488** |
| **45-55/65-75** | **-** |  | 0.0516 | 0.2348 | 0.9960 | 0.8322 |  | 0.7994 | 0.4859 | 0.1426 | 0.3919 |  | 0.9207 | 0.8127 | 0.9212 | 0.7080 |
| **45-55/>75** | **‡** |  | **0.0181** | 0.4427 | **0.0407** | **0.0327** |  | 0.2706 | 0.4679 | 0.3582 | - |  | 0.8901 | 0.3495 | 0.5885 | 0.1324 |
| **55-65/65-75** | **-** |  | 0.1200 | 0.1315 | 0.4139 | 0.4974 |  | 0.5071 | 0.8654 | 0.2565 | 0.4623 |  | 0.1297 | 0.1001 | 0.4119 | 0.1268 |
| **55-65/75<** | **⸸** |  | **0.0315** | 0.4023 | 0.0702 | **0.0411** |  | 0.5326 | 0.3669 | 0.5700 | - |  | 0.3806 | 0.7713 | 0.8578 | 0.3025 |
| **65-75/>75** | **#** |  | 0.2156 | 0.9327 | **0.0231** | **0.0237** |  | 0.3119 | 0.3927 | 0.0606 | - |  | 0.9355 | 0.2162 | 0.5270 | 0.1494 |

DAS28 ESR = erythrocyte sedimentation rate based DAS28 score; DAS28 CRP = C-reactive protein based DAS28 score; CDAI = Clinical Disease Activity Index score. V1: enrolment, V2: week 4 ± 2, V3: week 12 ± 2, EOS: End of Study. Significant differences between age subgroups are depicted in Figure 4 as follows: **p* < 0.05 at the DAS28 ESR as ‘<45’ < ‘>75’ at V1; ^‡^*p* < 0.02 at the DAS28 ESR as ‘45-55’ < ‘>75’ at V1; ^⸸^*p* < 0.05 at the DAS28 ESR as ‘55-65’ < ‘>75’ at V1; ^¤^*p* < 0.05 at the DAS28 ESR as ‘<45’ < ‘65-75’ at V2; **p* < 0.05 at the DAS28 ESR as ‘<45’ < ‘>75’ at V3; ^‡^*p* < 0.05 at the DAS28 ESR as ‘45-55’ < ‘>75’ at V3; ^#^*p* < 0.05 at the DAS28 ESR as ‘65-75’ < ‘>75’ at V3; **p* < 0.02 at the DAS28 ESR as ‘<45’ < ‘>75’ at EOS; ^‡^*p* < 0.05 at the DAS28 ESR as ‘45-55’ < ‘>75’ at EOS; ^⸸^*p* < 0.05 at the DAS28 ESR as ‘55-65’ < ‘>75’ at EOS; ^#^*p* < 0.05 at the DAS28 ESR as ‘65-75’ < ‘>75’ at EOS; ^§^*p* < 0.05 at the DAS28 CRP as ‘<45’ < ‘45-55’ at V2; ^$^*p* < 0.05 at the DAS28 CRP as ‘<45’ < ‘55-65’ at V2; ^¤^*p* < 0.05 at the DAS28 CRP as ‘<45’ < ‘65-75’ at V2; ^$^*p* < 0.005 at the DAS28 CRP as ‘<45’ < ‘55-65’ at V3; ^¤^*p* < 0.05 at the DAS28 CRP as ‘<45’ < ‘65-75’ at V3; **p* < 0.002 at the DAS28 CRP as ‘<45’ < ‘>75’ at V3; ^$^*p* < 0.05 at the CDAI as ‘<45’ < ‘55-65’ at EOS; ^%^*p* < 0.05 at the CDAI as ‘45-55’ < ’55-65<’ at EOS; *p* values are results of unpaired t-tests.

**Supplementary Table S4b. Distribution characteristics of the age subgroups.**

| **Age** |  |  |  |  |  |  |  |  |  |  |
| --- | --- | --- | --- | --- | --- | --- | --- | --- | --- | --- |
|  |  |  |  |  |  |  |  |  |  |  |
|  | **n** | **Mean** | **SE** | **SD** | **Median** | **Min** | **Max** | **CI(calc.)** | **CI(low)** | **CI(high)** |
|  |  |  |  |  |  |  |  |  |  |  |
| **ITT** | 333 | 57.505 | 0.646 | 11.785 | 59.280 | 20.876 | 82.543 | 1.270 | 56.235 | 58.775 |
|  |  |  |  |  |  |  |  |  |  |  |
| **<45** | 58 | 38.696 | 0.766 | 5.832 | 40.593 | 20.876 | 44.991 | 1.534 | 37.162 | 40.229 |
| **45-55** | 72 | 50.733 | 0.344 | 2.917 | 50.769 | 45.051 | 54.984 | 0.685 | 50.048 | 51.418 |
| **55-65** | 111 | 60.769 | 0.253 | 2.669 | 61.051 | 55.411 | 64.972 | 0.502 | 60.267 | 61.271 |
| **65-75** | 72 | 68.760 | 0.283 | 2.403 | 68.500 | 65.166 | 74.423 | 0.565 | 68.195 | 69.324 |
| **>75** | 20 | 77.800 | 0.494 | 2.210 | 77.389 | 75.083 | 82.543 | 1.035 | 76.765 | 78.834 |

Intention-to-treat population (ITT; n = 333) including all age subgroups [<45 = less than 45 years old, 45-55 = greater than 45 but less than 55 years old, 55-65 = greater than 55 but less than 65 years old, 65-75 = greater than 65 but less than 75 years old, >75 = greater than 75 years old; all at the time of V1]. SE = standard error; SD = standard deviation; n = number of patients in group; CI = confidence interval. The age of the patients (in years) was calculated with the following method: [date of informed consent (V1) (in days)]-[date of birth (in days)]/365.25; there were no patients exactly 55 or 65 years old.

**Supplementary Table S5a. Unpaired t-test results of all body weight subgroups at all visits.**

|  |  |  | **DAS28 ESR** | | | |  | **DAS28 CRP** | | | |  | **CDAI** | | | |
| --- | --- | --- | --- | --- | --- | --- | --- | --- | --- | --- | --- | --- | --- | --- | --- | --- |
|  |  |  |  |  |  |  |  |  |  |  |  |  |  |  |  |  |
| ***p*(T<=t) two-tail** |  |  | **V1** | **V2** | **V3** | **EOS** |  | **V1** | **V2** | **V3** | **EOS** |  | **V1** | **V2** | **V3** | **EOS** |
| **≤60/60-75** | **§** |  | 0.2287 | 0.6781 | 0.6020 | 0.6543 |  | 0.8878 | 0.0776 | 0.9062 | 0.7037 |  | 0.8226 | 0.4916 | 0.9957 | 0.9070 |
| **≤60/75-90** | **$** |  | 0.5712 | 0.7409 | **0.0164** | 0.2831 |  | 0.5899 | 0.8607 | 0.6904 | 0.9065 |  | 0.3930 | 0.9486 | **0.0074** | 0.5713 |
| **≤60/≥90** | **¤** |  | 0.1289 | 0.4795 | 0.3367 | 0.6649 |  | 0.9325 | 0.9167 | 0.8217 | 0.6244 |  | 0.1375 | 0.5663 | 0.0802 | 0.4764 |
| **60-75/75-90** | ***** |  | 0.6618 | 0.9345 | **0.0356** | 0.4173 |  | 0.4603 | 0.1359 | 0.5332 | 0.7843 |  | 0.4679 | 0.4700 | **0.0019** | 0.5895 |
| **60-75/≥90** | **%** |  | 0.6316 | 0.7161 | 0.5612 | 0.3763 |  | 0.7896 | 0.1806 | 0.7200 | 0.8219 |  | 0.1553 | 0.1999 | **0.0492** | 0.4692 |
| **75-90/≥90** | **‡** |  | 0.4193 | 0.6690 | 0.2483 | 0.1620 |  | 0.6036 | 0.9588 | 0.9009 | 0.6808 |  | 0.5959 | 0.6313 | 0.3063 | 0.9882 |

|  |  |  |  | | | |  |  | | | |  | **CDAI** | | | |
| --- | --- | --- | --- | --- | --- | --- | --- | --- | --- | --- | --- | --- | --- | --- | --- | --- |
|  |  |  |  |  |  |  |  |  |  |  |  |  |  |  |  |  |
| ***p*(T<=t) two-tail** |  |  |  |  |  |  |  |  |  |  |  |  | **V1** | **V2** | **V3** | **EOS** |
| **<75/≥75** | ***** |  |  |  |  |  |  |  |  |  |  |  | 0.1182 | 0.3427 | **0.0008** | 0.3760 |

DAS28 ESR = erythrocyte sedimentation rate based DAS28 score; DAS28 CRP = C-reactive protein based DAS28 score; CDAI = Clinical Disease Activity Index score. V1: enrolment, V2: week 4 ± 2, V3: week 12 ± 2, EOS: End of Study. Significant differences between body weight subgroups are depicted in Supplementary Figure S1 as follows: ^$^*p* < 0.02 at the DAS28 ESR as ‘≤60’ > ’75-90’ at V3; **p* < 0.05 at the DAS28 ESR as ’60-75’ > ’75-90’ at V3; ^$^*p* < 0.01 at the CDAI as ‘≤60’ > ’75-90’ at V3; **p* < 0.002 at the CDAI as ’60-75’ > ’75-90’ at V3; ^%^*p* < 0.05 at the CDAI as ’60-75’ > ’ **≥**90’ at V3; **p* < 0.001 at the CDAI as ’<75’ > ’ **≥**75’ at V3; *p* values are results of unpaired t-tests.

**Supplementary Table S5b. Distribution characteristics of the body weight subgroups.**

| **Body weight** | |  |  |  |  |  |  |  |  |  |
| --- | --- | --- | --- | --- | --- | --- | --- | --- | --- | --- |
|  |  |  |  |  |  |  |  |  |  |  |
|  | **n** | **Mean** | **SE** | **SD** | **Median** | **Min** | **Max** | **CI(calc.)** | **CI(low)** | **CI(high)** |
|  |  |  |  |  |  |  |  |  |  |  |
| **ITT** | 333 | 72.730 | 0.917 | 16.736 | 70.000 | 40.000 | 148.000 | 1.804 | 70.926 | 74.534 |
|  |  |  |  |  |  |  |  |  |  |  |
| **≤60** | 92 | 55.272 | 0.485 | 4.656 | 56.000 | 40.000 | 60.000 | 0.964 | 54.308 | 56.236 |
| **60-75** | 110 | 67.573 | 0.320 | 3.356 | 68.000 | 61.000 | 74.000 | 0.634 | 66.939 | 68.207 |
| **75-90** | 74 | 80.338 | 0.459 | 3.950 | 80.000 | 75.000 | 89.000 | 0.915 | 79.423 | 81.253 |
| **≥90** | 57 | 100.982 | 1.582 | 11.944 | 96.000 | 90.000 | 148.000 | 3.169 | 97.813 | 104.152 |

Intention-to-treat population (ITT; n = 333) including all body weight subgroups [≤60 = less than or equal to 60 kg, 60-75 = greater than 60 kg but less than 75 kg, 75-90 = equal to or greater than 75 kg but less than 90 kg, **≥**90 = equal to or greater than 90 kg; alternatively (Supplementary Figure S1C’, E’) <75 = less than 75kg, **≥**75 = equal to or greater than 75 kg; all measured at the time of V1]. SE = standard error; SD = standard deviation; n = number of patients in group; CI = confidence interval.

**Supplementary Table S6a. Unpaired t-test results of the gender subgroups at all visits.**

|  |  |  | **DAS28 ESR** | | | |  | **DAS28 CRP** | | | |  | **CDAI** | | | |
| --- | --- | --- | --- | --- | --- | --- | --- | --- | --- | --- | --- | --- | --- | --- | --- | --- |
|  |  |  |  |  |  |  |  |  |  |  |  |  |  |  |  |  |
| ***p*(T<=t) two-tail** |  |  | **V1** | **V2** | **V3** | **EOS** |  | **V1** | **V2** | **V3** | **EOS** |  | **V1** | **V2** | **V3** | **EOS** |
| **Male/Female** | **-** |  | 0.1184 | 0.7337 | 0.1766 | 0.1951 |  | 0.4322 | 0.3223 | 0.4821 | 0.9802 |  | 0.3190 | 0.5018 | 0.6603 | 0.7982 |

DAS28 ESR = erythrocyte sedimentation rate based DAS28 score; DAS28 CRP = C-reactive protein based DAS28 score; CDAI = Clinical Disease Activity Index score. V1: enrolment, V2: week 4 ± 2, V3: week 12 ± 2, EOS: End of Study. No significant differences were observed (Supplementary Figure 3).

**Supplementary Table S6b. Distribution characteristics of the gender subgroups.**

| **Gender** |  |  |  | **Age** |  |  |  |  |  |  |  |  |  |
| --- | --- | --- | --- | --- | --- | --- | --- | --- | --- | --- | --- | --- | --- |
|  |  |  |  |  |  |  |  |  |  |  |  |  |  |
|  | **n** | **%** |  |  | **Mean** | **SE** | **SD** | **Median** | **Min** | **Max** | **CI(calc.)** | **CI(low)** | **CI(high)** |
|  |  |  |  |  |  |  |  |  |  |  |  |  |  |
| **ITT** | **333** | **100.00** |  |  | 57.50494 | 0.645824 | 11.78519 | 59.27995 | 20.87611 | 82.54346 | 1.270423 | 56.23451 | 58.77536 |
|  |  |  |  |  |  |  |  |  |  |  |  |  |  |
| **Male** | 49 | 14.71% |  |  | 55.76739 | 1.701909 | 11.91336 | 57.49487 | 27.03901 | 76.64066 | 3.421917 | 52.34548 | 59.18931 |
| **Female** | 284 | 85.29% |  |  | 57.80473 | 0.696459 | 11.73694 | 59.38672 | 20.87611 | 82.54346 | 1.370898 | 56.43383 | 59.17562 |

Intention-to-treat population (ITT; n = 333); SE = standard error; SD = standard deviation; n = number of patients in group; CI = confidence interval.
